# Disordered eating among adolescents with chronic pain: the experience of a pediatric rheumatology subspecialty pain clinic

**DOI:** 10.1186/s12969-021-00506-4

**Published:** 2021-02-16

**Authors:** Lauren Pianucci, Maitry Sonagra, Brooke A. Greenberg, Diana R. Priestley, Sabrina Gmuca

**Affiliations:** 1 Arcadia University, Glenside, PA USA; 2 Division of Rheumatology, Department of Pediatrics, Children’s Hospital of Philadelphia, 3401 Civic Center Blvd, Wood Building First Floor, Philadelphia, PA USA; 3 PolicyLab, Children’s Hospital of Philadelphia, Philadelphia, PA USA; 4 Center for Pediatric Clinical Effectiveness, Children’s Hospital of Philadelphia, Philadelphia, PA USA; 5 Muhlenberg College, Allentown, PA USA; 6 The Pennsylvania State University, State College, PA USA; 7 Perelman School of Medicine, University of Pennsylvania, Philadelphia, PA USA

**Keywords:** Chronic pain, Eating disorders, Disordered eating, Adolescent health, Chronic musculoskeletal pain, Rheumatology

## Abstract

**Background:**

Disordered eating and chronic pain often co-occur in adolescents, but the relationship between these conditions is not well understood. We aimed to determine the prevalence of and to identify the clinical characteristics associated with the presence of disordered eating among adolescents with chronic musculoskeletal pain (CMP) presenting to a pediatric rheumatology subspecialty pain clinic.

**Methods:**

This was a retrospective cohort study of pediatric patients presenting to a pediatric rheumatology subspecialty pain clinic for an initial consultation from March 2018 to March 2019. We complemented data from an existing patient registry with secondary chart review for patients identified with disordered eating. We compared patient characteristics based on the presence or absence of disordered eating among adolescents with CMP. Logistic regression modeling was used to determine factors associated with disordered eating.

**Results:**

Of the 228 patients who were seen for an initial consultation in the pain clinic in 1 year, 51 (22.4%) had disordered eating. Only eight (15.7%) of the 51 patients identified with disordered eating had a previously documented formal eating disorder diagnosis. Through multivariate logistic regression modeling, we found that disordered eating was associated with older age, higher functional disability, presence of abdominal pain, presence of gastrointestinal comorbidities, and presence of anxiety (all *p* < 0.05).

**Conclusions:**

Adolescents with chronic pain, especially those who experience gastrointestinal issues, anxiety, and greater functional disability, should be evaluated for disordered eating by the treating clinician in order to ensure timely and appropriate treatment.

## Background

Emerging evidence suggests a relationship between disordered eating and chronic pain in adolescents [1–6]. A variety of factors may contribute to the development of disordered eating among youth with chronic pain. First, a sedentary lifestyle due to pain combined with the use of food as a coping mechanism for pain may result in weight gain and new concerns regarding body weight and body image [7–9]. Second, co-morbid mood disorders (e.g. anxiety and depression) may predispose youth to developing both chronic pain and eating disorders [6]. Third, chronic pain in the abdomen can lead to the development of disordered eating if the intake of food exacerbates pain-related symptoms [1, 10]. Fourth, mediating factors such as hormonal changes, cytokine derangements, and generalized hypermobility may result in both gastrointestinal issues and chronic pain [11–14]. However, chronic pain does not always precede the development of an eating disorder in youth who carry both of these diagnoses, as demonstrated by Sim et al. in their case-control study of adolescents with an eating disorder and chronic pain compared to youth with an isolated eating disorder [1]. Therefore, a full understanding of the relationship between adolescent chronic pain and disordered eating remains to be elucidated.

Disordered eating is defined as maladaptive eating and weight behaviors and attitudes that may not meet criteria for an eating disorder [15]. Eating disorders can be considered the most extreme form of disordered eating, but are not inclusive of all patients with disordered eating [16]. Patients who do not fit the diagnostic criteria for an eating disorder but display maladaptive eating behaviors or other symptoms can be considered to have disordered eating. While disordered eating is not an official diagnosis and does not have diagnostic criteria, the presence of disordered eating can lead to the development of eating disorders and other health complications. For this reason, it is important to identify signs and symptoms of disordered eating in clinical evaluations to allow for proper medical treatment [17].

A common non-inflammatory chronic pain condition affecting adolescents and young adults is chronic musculoskeletal pain (CMP) [18–20]. Adolescents with CMP can present with localized or diffuse pain, and a number of different body parts can be affected. Patients may, for example, have localized abdominal pain, localized pain in a limb, or total body pain. The general non-pharmacologic treatment approach for CMP includes intensive physical activity and cognitive behavioral therapy; patients receive specific treatment recommendations based on their symptom severity. Patients either get a referral for outpatient physical therapy (PT), occupational therapy (OT) and individual counseling; or an intensive pain rehabilitation program rooted in a multidisciplinary approach, including PT/OT, art therapy, music therapy, individual and group psychological counseling along with social work support [21, 22].

While prior research has demonstrated the prevalence of chronic pain among youth diagnosed with an eating disorder, no studies, to our knowledge, have examined the prevalence of eating disorders or disordered eating patterns among a population of youth with CMP. The main objective of this study therefore was to describe the prevalence of, and the clinical characteristics associated with, disordered eating among adolescents with CMP presenting to a pediatric rheumatology subspecialty pain clinic at a pediatric tertiary care center. A better understanding of risk factors for the development or presence of co-morbid disordered eating among youth with chronic pain would lead to improved screening measures as well as timely initiation of treatment for this potentially life-threatening condition.

## Methods

This was a retrospective cohort study of patients seen in a pediatric rheumatology subspecialty pain clinic at Children’s Hospital of Philadelphia (CHOP) from March 2018 to March 2019. All patients diagnosed with CMP by the treating medical provider during the study interval were included. The diagnosis of CMP was made according to The International Association for the Study of Pain definition of persistent or recurrent pain affecting the bone(s), joint(s), muscle(s), or related soft tissue(s) present for at least 3 months [18–20].

An existing IRB-approved longitudinal patient registry for the purposes of clinical research was used to identify patients with disordered eating. Details regarding this patient registry were previously published by Gmuca et al. [23]. Given this study was a retrospective chart review and patients were not screened for disordered eating, we a priori identified clinical variables and patient reported information to determine the presence of disordered eating. In this study, patients were identified as having disordered eating if at least one of the following applied: patient reported modifying or restricting their diet due to pain; patient reported pain related to appetite changes; patient or parent reported or clinician identified (through medical record and growth curve) pain related weight loss; patient or parent reported stress about body image/weight/food; vomiting or diarrhea of unknown origin occurred regularly following meals; history of diagnosed eating disorder; reported stress/binge/compulsive eating; unexplained feeding tube placement; or malnutrition within 3 years of initial clinic consultation. Patients were identified as not having disordered eating if any of the following applied: absence of inclusion criteria; healthy weight and no eating changes; history of malnutrition was resolved at least 3 years prior to presentation in clinic; or patient had an oncologic diagnosis.

The existing patient registry includes a variety of clinical variables and patient-reported outcome measures such as the functional disability inventory (FDI) which is a 15-item measure assessing pain-related disability [24–27]. Data in the existing patient registry was complemented by retrospective chart review to abstract additional information on the cohort of patients with disordered eating. Additional clinical data including pain symptoms, history of eating disorder, weight classification, and self-reported psychological history were collected from the rheumatologists’ and psychologists’ assessments for each patient’s initial consultation. All records were reviewed by two researchers to ensure accurate data collection. Data abstracted from the medical records of patients who were deemed positive for disordered eating were entered into a secure, password protected, standardized REDCap (Research Electronic Data Capture) data entry form. This study received approval from the overseeing Institutional Review Board.

Data from the existing patient registry for all patients were merged with the data abstracted for patients with disordered eating to create a comprehensive dataset for all descriptive statistics and analyses. Patient demographics and clinical characteristics were summarized by median and interquartile range for continuous variables and frequency and percentage for categorical variables. Variations in characteristics between those with disordered eating and those without disordered eating were analyzed using chi-square and Fisher’s exact tests as appropriate for categorical variables and Wilcoxon rank-sum tests for continuous variables. *P*-values less than 0.05 were considered statistically significant. Bivariate analysis was performed for all variables with potential to influence the presence of disordered eating. Variables found to be significantly associated (*p* < 0.05) on bivariate analysis were used to perform multivariate logistic regression modeling. Multivariate logistic regression modeling was performed using backwards stepwise selection with a significance cut-off of *p* = 0.10. Variables included in the selected model due to significance were used to produce the final logistic regression model. All analyses were performed using Stata 15.1.

## Results

Of the 228 patients who were seen for an initial consultation and diagnosed with CMP in a pediatric rheumatology subspecialty pain clinic in a one-year period, 51 (22.4%) had disordered eating. The total cohort was predominantly female (80.3%) and Caucasian (77.6%). The median age of patients was 14 years (IQR 12–15.5). The mean duration of pain symptoms prior to diagnosis of CMP was 18 months (IQR 8–36).

We compared demographics and clinical characteristics between patients with and without disordered eating, as shown in Table 1. Patients with disordered eating were slightly older at presentation than patients without disordered eating (median age 15 years [IQR: 13–15] vs. 14 years [IQR: 12–15]; *p* < 0.01). Patients with disordered eating were more likely to have a higher widespread pain index (WPI) (median 7 [IQR: 3–12] vs. 5 [IQR: 1–9]; *p* = 0.03) and symptom severity score (SSS) (median 7 [IQR: 5–9] vs. 6 [IQR: 3–7]; *p* < 0.001) [28]. Symptoms of abdominal pain, total body pain, fatigue, and dizziness were more common among patients with disordered eating (all *p* < 0.05). The median self-reported energy level (rated from 0 to 100) was lower among patients with disordered eating (median 50 [IQR: 33–75] vs. 70 [IQR: 50–87]; *p* < 0.01). Patients with disordered eating had higher functional disability inventory (FDI) scores than those without (median FDI score of 33 [IQR: 20–39] compared to 23 [IQR: 14–31]; *p* < 0.001) [24–27]. Patients with disordered eating received more medical interventions prior to being seen in the pain clinic (all *p* < 0.05). Psychological and physical comorbid conditions were more common among patients with disordered eating (Table 1; all *p* < 0.05).

**Table 1:** Patient demographics and clinical characteristics (*N* = 228)

Characteristic	No disordered eating (*n* = 177)	Disordered eating (*n* = 51)	*P* value
**Demographics**, n (%)			
Female	141 (79.7)	42 (82.4)	0.67
Age	14 (12–15)	15 (13–16)	**< 0.01**
Race			
White	138 (78.0)	39 (76.5)	0.80
Black	10 (5.7)	4 (7.8)	
Other	29 (16.4)	8 (15.7)	
**Clinical Characteristics**, n (%); median (IQR)			
Duration of symptoms (months)	12 (7–36)	24 (12–36)	0.25
Pain frequency			
Intermittent	27 (15.3)	6 (11.8)	0.65
Constant	138 (78.0)	44 (86.3)	
Intermittent and constant	9 (5.1)	1 (2.0)	
Trigger event	68 (38.4)	14 (27.5)	0.15
BMI (kg/m^2^)			
Underweight (<5th percentile)	2 (1.2)	3 (6.0)	0.08
Normal weight (5th- <85th percentile)	93 (54.1)	27 (54.0)	0.99
Overweight (85th- <95th percentile)	35 (20.4)	7 (14.0)	0.31
Obese (≥95th percentile)	42 (24.4)	13 (26.0)	0.82
Widespreadness of pain (WPI)	5 (1–9)	7 (3–12)	**0.03**
Symptom severity score	6 (3–7)	7 (5–9)	**0.001**
Beighton Score (0–9)	0 (0–2)	0 (0–2)	0.96
**Patient Reported Outcomes**, n (%), median (IQR)			
Allodynia	152 (85.9)	45 (88.2)	0.67
Attending school full time	117 (66.1)	22 (43.1)	**< 0.01**
Fatigue	81 (45.8)	33 (64.7)	**0.02**
Energy level (0–100%)	70 (50–87)	50 (33–75)	**< 0.01**
Abdominal pain	65 (36.7)	38 (74.5)	**< 0.001**
Dizziness	63 (35.6)	26 (51.0)	0.05
Pain (VAS)	60.5 (36–73)	67.5 (40–73)	0.27
Total body pain	43 (24.3)	21 (41.2)	**0.02**
Functional disability (FDI)	23 (14–31)	32.5 (20–39)	**< 0.001**
Pain made worse by eating	4 (2.3)	6 (11.8)	**0.01**
**Medical Interventions**, n (%)			
Evaluated by gastroenterologist	46 (26.0)	22 (43.1)	**0.02**
Endoscopy	28 (15.8)	16 (31.4)	**0.01**
Colonoscopy	20 (11.3)	11 (21.6)	0.06
Upper gastrointestinal study	17 (9.6)	13 (25.5)	**< 0.01**
Evaluated by dietician	10 (5.7)	11 (21.6)	**0.001**
**Medical and Psychological Comorbidities**, n (%)			
Anxiety	78 (44.1)	34 (66.7)	**< 0.01**
Depression	47 (26.6)	25 (49.0)	**< 0.01**
Suicidality	26 (14.8)	22 (44.0)	**< 0.001**
Conversion disorder	11 (6.3)	2 (4.0)	0.41
Constipation	3 (1.7)	2 (3.9)	0.31
Reflux/gastroesophageal reflux disease (GERD)	3 (1.7)	6 (11.8)	**< 0.01**
Inflammatory bowel disease (IBD)	2 (1.1)	4 (7.8)	**0.02**
Irritable bowel syndrome (IBS)	0 (0.0)	6 (11.8)	**< 0.001**

*IQR* Interquartile range, *BMI* Body mass index (*N* = 222), measured in kg/m^2^; Duration of symptoms (*N* = 225); Trigger event defined as patient reported illness, injury, trauma, or surgery that occurred at or closely prior to the time of pain onset; *WPI* Widespread pain index (0–19), measure of widespreadness of pain; Symptom severity score (0–12) (*N* = 224), measure of condition severity; WPI and SS score defined according to the 2010 College of Rheumatology Criteria for Fibromyalgia Syndrome [28]; Beighton score (0–9), measure of joint hypermobility, score of ≥6 indicates hypermobility (*N* = 208); Energy level (*N* = 223); *VAS* Visual analog scale (*N* = 220), rating of level of pain (0–100) (for patients missing VAS in database, verbal reported pain score (0–10) was multiplied by 10 to supplement missing VAS); *FDI* Functional disability inventory (*N* = 224), measure of disability (0–60), with higher scores indicating more functional disability [24–27]; Suicidality (*N* = 226); Conversion disorder (*N* = 224). Of the entire cohort of patients with CMP, 126 patients were diagnosed with diffuse amplified musculoskeletal pain syndrome (AMPS), 76 were diagnosed with localized AMPS, and 36 were diagnosed with complex regional pain syndrome

We further classified patients with disordered eating based on additional characteristics that were abstracted from the medical records. Patients with disordered eating commonly reported that pain caused poor appetite (72.6%), changes in eating behaviors (72.6%), and weight loss (41.2%). Some patients with disordered eating reported stress over body shape (31.4%), weight (23.5%), and food (13.7%). Most patients with disordered eating (86.3%) reported at least one of the following gastrointestinal (GI) symptoms: nausea (52.9%), vomiting (34.5%), pain associated with eating (29.4%), constipation (25.5%), eating/appetite problems (17.7%), diarrhea (13.7%), and acid reflux/heartburn (9.8%). A diagnosis of at least one GI condition (GERD, gastroparesis, IBS, IBD, constipation, Hirchsprung’s disease, small bowel bacterial overgrowth) existed for 27.5% of patients, but only four patients (7.8%) were taking one or more GI medications. Of the 51 patients with disordered eating, only eight (15.7%) had received a formal diagnosis of eating disorder as per the medical history record prior to the initial consultation in the pain clinic. All eight patients with an eating disorder diagnosis had previously received formal inpatient or outpatient eating disorder treatment. Recommendations made at the initial consultation for patients with disordered eating included bloodwork/lab tests (13.7%), referral to another medical subspecialist for further evaluation (13.7%), and bone density scan (9.8%).

In logistic regression modeling, we found that patient age (OR 1.21 [95% CI 1.04–1.40]), FDI score (OR 1.05 [95% CI 1.02–1.08]), presence of abdominal pain (OR 5.04 [95% CI 2.50–10.14]), GI comorbidities (OR 7.47 [95% CI 2.76–20.21]), and anxiety (OR 2.54 [95% CI 1.32–4.88]) were associated with increased odds of disordered eating in bivariate logistic regression analysis. All variables associated in bivariate analysis remained significantly associated with disordered eating in the multivariate logistic regression model (Table 2).

**Table 2:** Predictors of the presence of disordered eating among youth with chronic musculoskeletal pain

Factor	Bivariate Analysis		Multivariate Analysis	
	OR	95% CI	OR	95% CI
Abdominal pain	5.04	2.50–10.14	3.33	1.55–7.15
Age	1.21	1.04–1.40	1.29	1.06–1.56
Anxiety	2.54	1.32–4.88	2.20	1.03–4.73
Functional disability (FDI)	1.05	1.02–1.08	1.05	1.01–1.08
GI comorbidities	7.47	2.76–20.21	7.44	2.29–24.23

*FDI* Functional disability inventory, measure of disability (0–60) with higher scores indicating greater pain-related functional disability [24–27]; GI comorbidities defined as presence of at least one of the following (patient reported or clinician diagnosed): inflammatory bowel disease (*IBD*), irritable bowel syndrome (*IBS*), gastrointestinal reflux, or constipation

## Discussion

In a cohort of patients (*N* = 228) presenting to a pediatric rheumatology subspecialty pain clinic for initial consultation in one calendar year, nearly one quarter (*n* = 51) were identified as having disordered eating. Only a minority (15.7%) of the cohort identified to have disordered eating (*n* = 51) had previously received a diagnosis of a formal eating disorder. We found that disordered eating was associated with older age, greater functional disability, and the presence of abdominal pain, GI comorbidities, and anxiety among adolescents with CMP. Attention to these clinical characteristics in patients seen in subspecialty pain clinics can help clinicians identify adolescents with CMP who are potentially at risk for disordered eating.

The results of our analyses align well with the current literature. Our cohort had a median age of 14 years and was predominantly female (80.3%) and Caucasian (77.6%). This is analogous to the characteristics of a typical patient hospitalized for eating disorders or somatoform disorders (adolescent, white female) [29]. We found that 22.4% of our cohort had disordered eating, corroborating previous findings that chronic pain and disordered eating commonly co-occur in adolescents [1–6].

Among the study subjects identified with disordered eating, patients often reported loss of appetite (72.6%), changes in eating behaviors (72.6%), and weight loss (41.2%) due to pain, which is in line with previous studies in both adolescents and adults [2, 8]. Furthermore, eating disorders often co-occur with mood disorders [6]. We correspondingly determined that the prevalence of anxiety (66.7%), depression (49.0%), and suicidality (44.0%) in patients with disordered eating was significantly higher than in those without (all *p* < 0.01).

Furthermore, our findings that patients with disordered eating more commonly have symptoms of fatigue (*p* = 0.02) and lower median self-reported energy (*p* < 0.01) support previous evidence that disordered eating and fatigue commonly co-occur [1, 4, 10]. We additionally showed that patients with disordered eating have higher SSS and higher FDI (both *p* < 0.001) than those without. Patients presenting in pain clinics with abdominal or widespread chronic pain, greater overall symptomatology, and increased disability should therefore be considered high risk for disordered eating.

In addition to supporting previous findings, we add new knowledge that has potential to improve identification of disordered eating in adolescents with CMP. Previous studies in adolescents have found an association between disordered eating and pain location, specifically abdominal pain and headache [1, 3, 4, 30]. Our evidence does indicate that patients with CMP and co-morbid disordered eating are more likely to report abdominal pain (*p* < 0.001) than those without disordered eating. We additionally found that CMP patients with disordered eating more commonly report total body pain (*p* = 0.02). This study is the first to identify disordered eating in a cohort of pediatric chronic pain patients specifically diagnosed with CMP. This may explain why the association between full body pain and disordered eating was not identified previously.

Our findings are also unique as we identified all patients with disordered eating, rather than limiting our analyses only to those with a formal eating disorder diagnosis. Only 15.7% of patients with disordered eating had a formal eating disorder diagnosis prior to their initial consultation; this is noteworthy, as many patients we identified as having disordered eating are likely being overlooked and not receiving additional support and treatment for disordered eating. By identifying these patients as having disordered eating upon their evaluation for chronic pain, we can ensure that patients receive timely and appropriate care for their disordered eating prior to initiating CMP treatment. The non-pharmacologic treatment for CMP involves intensive physical activity; it is therefore critical that these patients have adequate caloric intake and weight stabilization [21].

The generalizability of this study is limited as the study population was from a single, highly specialized clinic for children with chronic pain. This patient population may not be representative of all children with CMP and other chronic pain conditions. Our clinic may experience referral bias of the most severe cases of CMP due to its nature as a specialized clinic at a tertiary care hospital. This bias may have led to an overestimate of the extent of disordered eating in this population compared to all pediatric chronic pain patients. Despite this possible overestimation, this study’s findings have noteworthy implications for clinicians treating pediatric patients with chronic pain in a variety of settings.

The retrospective nature of this study may have resulted in an ascertainment bias by limiting our ability to detect all patients experiencing disordered eating. Data included in the patient registry was not designed specifically with our research question in mind and we do not routinely administer disordered eating screening questionnaires in our clinic. Therefore, data for some variables of interest were limited, but this was mitigated by re-abstraction of data from the medical record for patients identified as having disordered eating. In addition, some variables used to classify disordered eating, such as pain related weight loss, were based on patient/parent reported information and/or clinician identified objective information. Our evaluation of these variables was limited by our inability to distinguish between objective and subjective information.

Despite these limitations, this study provides an important contribution to the existing knowledge on the relationship between disordered eating and CMP in adolescents. This study complements previous work [1] by identifying the characteristics of patients prioritizing pain in medical care. Clinicians evaluating and treating adolescents with CMP should consider the factors we have identified to be associated with disordered eating in their evaluations. In addition, our findings indicate the potential benefit of including a dietician or nutritionist in the multidisciplinary CMP treatment team. Patients are currently referred to dieticians or nutritionists when appropriate, but integration of such services into the comprehensive treatment of CMP in our clinic could streamline the evaluation and treatment of disordered eating and other nutritional issues. Future work to identify the feasibility and potential impact of such an addition to the multidisciplinary treatment team is warranted.

## Conclusions

We identified the characteristics of patients with disordered eating in a cohort of patients presenting for evaluation of chronic pain in a pediatric rheumatology subspecialty pain clinic. Our findings indicate that adolescents with chronic pain, especially those who experience gastrointestinal issues, anxiety, and greater functional disability, should be evaluated for disordered eating by the treating clinician in order to ensure timely and appropriate treatment. Future studies to conduct systematic screening of patients presenting to the pediatric rheumatology subspecialty pain clinic and other pain clinics would help to determine if formal screening for disordered eating would be beneficial for this population.

## Data Availability

The dataset used and/or analyzed during the current study are available from the corresponding author on reasonable request.

## Abbreviations

CMP: Chronic musculoskeletal pain
FDI: Functional disability inventory
GERD: Gastroesophageal reflux disease
GI: Gastrointestinal
IBD: Inflammatory bowel disease
IBS: Irritable bowel syndrome
VAS: Visual analog scale
WPI: Widespread pain index
